# Iowa State Veterinary Medical Association

**Published:** 1896-01

**Authors:** John E. Brown

**Affiliations:** Secretary


### SOCIETY PROCEEDINGS.

### IOWA STATE VETERINARY MEDICAL ASSOCIATION.

(Concluded from page 767, November, 1895.)

AFTERNOON SESSION, SEPT. 9, 1895, 1.30 P.M.

President Miller called meeting to order, and appointed Drs. Edwards, Kingery, and McBirney to vacancies on Board of Censors.

Report of Committee on Publication of the proceedings of the 1894 meeting was made by Dr. Brown, chairman, who reported that the same had been printed in pamphlet form, paper cover, 134 pages, at fifty cents per page, 250 copies. Total cost, $67.00, and a copy distributed to each member of the Association. On motion of Dr. Niles, seconded by Dr. Gibson, the report was accepted.

Report of committee to secure the 1895 annual meeting of the U. S. V, M. A. in Iowa, was made by the Chairman, Dr. Niles, who said the committee, after long and continued efforts, had secured the meeting; that it will convene in the Horticultural Rooms at the Capitol Building, tomorrow, and promises to be a great success. On motion duly made and seconded, a vote passed to accept the report.

The Secretary, Dr. John E. Brown, offered the following report:

"In reviewing the work of this Society for the past year, we become cognizant of the fact that our Association has just experienced an era of special prosperity. First—probably not since its organization has the Society held such an interesting, and in every sense profitable, convention as the one held last year. The members there present, filled with enthusiasm, have spread the news of its success in all sections of the State. Following those reports went the official report, a pamphlet of 125 pages, to each member, giving as full an account of the proceedings as your Secretary was able to note during the sessions. From the many complimentary notices received, I am sure this effort has been appreciated by our members, and that it has proved to be quite an active agent in arousing dormant interests among members not present, in the Association work. As to the wisdom of issuing such a report each year I think there can be no question. Through it, members unavoidably absent are kept in sympathetic touch with the aims and endeavors of the Society. The publication of such a report, however, involves an expenditure of money that our Association with its present rate of dues will not be able to meet. It will be found by reference to our financial statements in the past that, with our present membership and mailing list, the regular yearly expenses will not fall far short of from $50 to $65. Add to that $60 to $70 for the printing of the annual report and we have a total of, say, $125. Our present membership being seventy-one, this equals a per capita of about $1.75. From this there would be some shrinkage in the form of lapses and suspensions; but new membership fees each year would probably balance that deficit. From the above statement, one of two things becomes painfully evident: we must either raise our annual dues or cease to issue our report.

"Another crowning feature in the work of the Association during the past year was the successful effort of our committee to capture and bring to Iowa the annual meeting for 1895 of the U. S. V. M. A. This puts in reach of every veterinarian in the State an opportunity to attend what now promises to be one of the most brilliant and profitable conventions of the character ever convened.

"With the increase of the veterinary population of Iowa, now numbering 165, the I. S. V. M. A. has added a very creditable percentage to its membership. Last year 17 new members were admitted, and at this session several applicants seek admission. All of us who have been engaged in a private practice know that for the past two years the business has been on a general decline. Under these circumstances, feeling that the future was uncertain, many have become somewhat discouraged and held off that would have under favorable circumstances joined the Association and become useful members. Not only has that cause played an important part in lessening the number of applicants for membership, but has also created a disinterested spirit in many of our older members, keeping them away from our meetings, thus weakening our forces and crippling our progress. Our present membership is made up of 71 active and 7 honorary members. On November 14, 1894, the date of our last meeting, there was outstanding against the members $181 in unpaid dues and memberships; $94 of this was cancelled with the suspensions directed at that time, leaving $87 against the membership then in good standing. Your Secretary was instructed at that meeting to notify each of those whose names were proposed for suspension for non-payment of dues of his indebtedness to the Association, and that if the amount named was not immediately paid each one so failing to remit should stand suspended. Notices to the above effect were mailed on or about December 15, 1894, and only one reply ever reached this office.

"Both through circular letters and private correspondence I have endeavored to keep in touch with, not only our own membership, but with all members of the profession in Iowa. A close watch has been kept for new representatives of the profession coming into the State, and no effort has been spared to enhance the interests of the Society.

"During the time that I have served as your Secretary I have endeavored to compile a complete list of the names of all graduate veterinarians practising in the State, together with the post-office address, the name of the college of which they are graduates, and the year of graduation. This list has required frequent revisions, and, while it may not now be entirely complete, by its aid I have been in communication with almost every graduate in Iowa. There is not a man in Iowa, to my knowledge, who is eligible to membership in the I. S. V. M. A. who has not only been urged to join the Association, but was also given official notice of this meeting and invited to attend.

"The report of the 1894 meeting was received from the printers on June 11th, and on June 12th a copy was mailed at the Oskaloosa postoffice to each member of the Association, except two or three whose postoffice address had been changed and I had some trouble in locating. Complimentary copies were sent to the *Veterinary Review*, *Veterinary Magazine*, and JOURNAL OF COMPARATIVE MEDICINE AND VETERINARY ARCHIVES, with a notice that duplicate copies might be obtained from the Secretary for fifty cents each. Similar notices with sample pages and programme of the meeting were mailed to members of the profession in this and other States. Orders came in sparsely, and complimentary copies were then sent to some of the stock journals with the same notice, but the pamphlets did not meet with ready sale, only a few copies were called for.

"The 'resolutions' adopted at the last meeting and directed to the State Board of Health, were sent (one copy) to the President, Dr. J. C. Shrader, and (one copy) to the Secretary. Responding to these resolutions, Dr. Shrader sent me the following letter:

DR. JOHN E. BROWN, Sect. S.V.M.A., Oskaloosa, Iowa.

DEAR DOCTOR: Yours containing copy of resolutions passed by your Association in relation to tuberculosis in cattle duly received. I am pleased to note this action taken in regard to so important a matter. I shall take pleasure in laying this before the Board at their next meeting, about February 1st. Allow me to assure you the Board are awake to the great danger to our people from this source, but our hands are almost tied for lack of funds to thoroughly investigate and inspect the herds of our State, but we will do all we can.

Truly yours,

IOWA CITY, IOWA, January 17, 1895. J. C. SHRADER.

"The resolution adopted and directed to the Iowa Legislature is still in my possession, as that body has not been in session since our meeting.

"All will now agree that the past year's work is well marked with happy results. Certainly the I. S. V. M. A. is an potent factor for the elevation and advancement of veterinary interests in Iowa. We, as veterinarians, have failed in great part in the past to present ourselves and the profession to the public in that light which would suggest to them that we might be a necessity or even a possible help in providing for and enforcement of sanitary measures, the now most important part of all veterinary work; and until the public recognizes the profession in some other capacity, as well as simply treating a few of the ills and injuries of the lower animals, we will not find ourselves occupying a plane, professionally, very much in advance of the common country "horse-doctor." The I. S. V. M. A. has all these years had for its high aim the part of keeping its members up to the standard of proficiency. I do not believe the Association has accomplished all that it might have, but if the profession has not given it universal support then the blame rests heavily upon those who have failed to do their part. I believe the Association can and will do more in the future than it has in the past. I believe more of our members should be drafted into a working force. The profession is in its infancy. Investigators are constantly revealing hitherto hidden facts. New surgical operations are being performed and new medicinal agents employed with greater or less success. Some of our sister societies have created committees to investigate these and make reports at their regular meetings. Would it not be well for us to follow their example? That Iowa has a live organization whose members generally are well abreast of the times, and that they as a Society are a power in the advancement of the veterinary interests of the State, are facts in which each member may glory in a just sense of pride. Meeting together at these conventions, with a free interchange of thought and ideas, invariably aids one to realize his own professional narrowness, and proves to be a wholesome stimulus to a healthful effort for greater achievements.

"In closing I wish to thank the members of the Association for their hearty co-operation and ready response to all calls, thus greatly aiding in the work of this office. With a sincere hope that the coming year will be one of renewed activity and greater prosperity to our beloved profession and to the I. S. V. M. A., I respectfully submit the foregoing as my official report."

On motion of Dr. Austin, seconded by Dr. Bown, a vote passed to accept the report. Discussions followed regarding raising of dues and printing of reports. It was not deemed expedient to increase the dues, but, as it is desirable to have a report of the meeting, Drs. Stuart and Niles were appointed a committee of two to confer with the publishers of THE JOURNAL OF COMPARATIVE MEDICINE AND VETERINARY ARCHIVES and attempt to have the proceedings of the meeting published in that journal and secure a reprint for each member of the Association.

The Treasurer's report showed that during the period of time from November 14, 1894, to September 9, 1895, the total receipts were $113.75, and expenditures $123.93. On motion of Dr. Johnson, seconded by Dr. Parslow, the report was referred to the Auditing Committee. Drs. Scott, Bown, and McBirney were appointed an auditing committee.

Dr. Stalker, Chairman of the Committee on Newspaper Articles, said the committee had done nothing, and he was not advised as to the duties of that committee. A discussion followed by Drs. Miller, Gibson, Niles, and others. The committee was continued, its duties to be general supervision over all articles relating to sanitation, legislation, and education.

The Auditing Committee reported the accounts of the Treasurer in satisfactory condition, and the report was accepted.

The Chairman of the Committee on United States Army Legislation was not present, hence no report was made. On motion of Dr. Niles, with Dr. Gibson's second, a vote was passed continuing the same committee another year.

The Board of Censors reported favorably on the following applicants for membership and the same were elected: Drs. C. A. Clinton, M.D.C.; H. M. Gillian, V.S.; G. E. Armstrong, M.D.C.; Charles Williams, D.V.S.; Peter Malcolm, V.S.; James Vincent, D.V.M.; S. T. Miller, D.V.S.; H. T. Stewart, M.D.C.; D. H. Miller, M.D.C.; H. E. Talbot, M.D.C.; S. K. Hazlet, M.D.C.; C. E. Stewart, M.D.C.; J. L. Williamson, D.V.S.; A. Collasawitz, M.D.C.

To accommodate the time of one of the essayists, the business of the meeting was now set aside, and the literary part of the programme inserted.

Dr. S. H. Kingery, of Creston, read a very interesting and carefully prepared paper on "Tetanus."[1]

The discussions following were taken part in by Drs. Carey, Niles, Gibson, Wattles, Brown, Hoskins, Whitbeck, Walrod, Miller, Williams, Edwards, and others. Quite a general impression seemed to prevail that we do not have idiopathic tetanus; that it is due to traumatic causes, though in many cases the traumatism is obscure. Also that recovery depends more on the

[1] See page 27.

severity of the case than any special line of treatment. Quietude, darkness, and non-interference still have their advocates, while others entirely disregard these precautions and really advise the reverse, leaving the animal to his accustomed habits. Serum-therapy was also discussed.

Dr. W. L. Williams, of Bozeman, Mont., was then called upon and read an excellent paper entitled "Immobility in the Treatment of Diseases of the Extremities."[1]

The discussion of "splints" of various kinds was indulged in by Drs. Stewart, Vincent, Gibson, Kingery, Carey, and others. The meeting adjourned till 7.30 P.M.

EVENING SESSION, 7.30 P.M.

Meeting called to order by President Miller. Dr. Stalker, Chairman of Committee on Arrangements for U. S. V. M. A. Meeting, said that the Horticultural Rooms at the Capitol Building had been secured for the place of meeting; that the city Mayor or Governor Jackson would be present at the opening session and give an address of welcome. The Savery House had been selected as headquarters. Entertainment for the visitors had been talked over, but nothing decided upon. A trip to Ames College was talked of; also a half-day's visit at the State Fair, but the officers of the United States Association object to either, on account of consuming too much valuable time. After considerable discussion on the subject, the committee was instructed to arrange for a theatre party, and each member of the Society was assessed one dollar to cover the expenses of the same. Dr. Wake was appointed to collect the assessment from the members present, the money to be turned over to the chairman of the committee.

The report of the Committee on Veterinary Legislation (Drs. M. Stalker and John E. Brown) was made by the Chairman, Dr. M. Stalker, as follows:

"A bill, a copy of which is herewith presented, was prepared and introduced in the last general assembly. It was referred to the Senate Committee on Medicine and Surgery, and received the consideration of that committee. The opposition to any measure regulating the title, or practice of veterinary surgeons, without conferring diplomas on non-graduates, was such as to make it manifest to the friends of the bill that it could not pass without being so amended. It was the judgment of the committee, and it is our belief from discussions had in this body over the same subject, that it is the judgment of the entire Association that any bill which provides for issuing diplomas to non-graduate practitioners would be highly detrimental to the advancement of veterinary science in the State. It was our judgment that any benefits resulting from desirable provisions of the bill would be more than neutralized by such an amendment. Your committee was left to choose between withdrawing the bill and accepting an amendment that in their judgment would render it worse than no legislation. They choose the former course, and asked to have the bill withdrawn."

Discussions followed by many of the members. Dr. Johnson moved and Dr. Bown seconded that the report be accepted. Moved by Dr. Niles, with Dr. Bown second, to amend by adding, "and the committee retained in the interest of the bill." Vote on the motion as amended was carried.

[1] See page 703, November, 1875.

Report of Committee on Sanitation (Drs. W. B. Niles, W. H. Austin, and G. A. Johnson) was made by the Chairman, Dr. Niles, and read as follows:

"When last year I suggested the advisability of appointing a committee on sanitary science, the thought did not occur to me that I might be placed on the committee. Since our worthy President has seen fit to make me chairman of that committee, I find it necessary to present something for your consideration.

"The field of sanitation is so large that your committee has found it a difficult matter to determine how best to deal with the subject. After consulting with other members, it was decided that we would endeavor to learn of what was being done along this line in some of the more progressive European countries and in the different States of our own country, and discuss what we are doing in Iowa, and why we should do more; limiting the discussion to a consideration of dangers arising from the use of animal foods, and the restriction of contagious diseases of our domestic animals. Being disappointed in obtaining copies of the sanitary laws of European countries, we will only state regarding this point what is probably already known to most of you, that these countries, being older and so governed that compulsory laws can be more easily passed and enforced, have in operation very efficient sanitary rules and regulations that not only tend to arrest and prevent the spread of contagious diseases, but to also reduce to a minimum the dangers arising from the use of animals and their products for food. This is notably the case in Germany, where a very thorough system of meat-inspection is in vogue. All animals must be killed at public abattoirs and carefully inspected before being placed on the market. I have heard it stated that at least in one country of Europe all animals, whether for public or private use, must be killed and inspected at the abattoir. This is no doubt true.

"In our own country quite different conditions prevail. We are a new country and do not take kindly to compulsory laws of any kind. Nevertheless, we have made much progress during the last ten years.

"The National Government, by establishing the Bureau of Animal Industry with a veterinarian as chief, has accomplished very much in the way of preventing the spread of disease in domestic animals, and by the establishment of quarantine rules and regulations set an example to the several States. As a result of this and other causes a majority of the States have passed laws for the purpose of preventing the spread of contagious and infectious diseases.

"You are all familiar with our own State law, which provides for the appointment of a State veterinarian and gives him general supervision of all contagious and infectious diseases among domestic animals within, or that may be in transit through the State, with power to establish quarantine, etc. Several other laws relative to diseases of animals are also on our statute books.

"The sanitary laws of other States are quite similar to our own. By the enactment of these laws in the several States very much has been done, as we all know, in limiting the extent of infectious and contagious diseases among our farm animals. In some States glanders has been almost eradicated, Texas fever largely rendered a thing of the past, and contagious pleuro-pneumonia, by the assistance of the National Government, absolutely stamped out of the United States. Many other affections have also become more or less restricted in their ravages. Nothwithstanding all this, we believe sanitary work along this line can be much improved, as we shall attempt to show later on.

"In another field of sanitary work, that of lessening the danger arising from the consumption of diseased foods derived from animal sources, we have not progressed so far. Until recently only in a few of our largest cities was there any system of meat-inspection in vogue. About four years ago the National Department of Agriculture inaugurated the work at some of the large packing-houses engaged in exporting meat to Europe. This work on the part of the department has been extended until now many of these houses are provided with veterinary inspectors, and it is with pleasure that we note the continued improvement in the work. All cattle going through houses having inspectors are now inspected both on foot and after slaughter, whether intended for export or home consumption. In addition to the work already mentioned a few cities employ a veterinarian, whose duty it is to inspect all meats sold in the city as well as fish, fruits, etc. Thus we see that something is being done toward providing the public with wholesome animal food, but that as yet only a small fraction of the meat consumed in the country is inspected in any way, To illustrate, only at two points in our own State have we government meat-inspectors, and only one city has city inspection of meats. The condition in other States is very similar. It is pleasing to note, however, that very recently veterinary inspectors have been appointed in several large cities of the country. At St. Louis outbreaks of Texas fever and anthrax emphasized to the inhabitants the necessity for careful inspection of meats and dairies.

"We all know that during the past two years the public has been fast learning the importance of sanitation, and that never before have the members of our profession realized as much as now the importance of a knowledge of this subject. It is also pleasing to note that many of our veterinary colleges have realized the importance of the subject and have added meat and dairy inspection to the course of study.

"Let us more fully consider the subject as it concerns us here. It may be asked, What contagious diseases of animals occur in this State calling for the application of sanitary regulations? Several may be mentioned—glanders, while rarely seen compared with ten years ago, still demands attention. There is no question that this affection could be completely eradicated could we have and enforce the necessary regulations. Some recent work in Europe has shown that many latent cases of this disease exist in exposed herds, only capable of being detected by the use of mallein. These obscure cases escape detection and eventually start new outbreaks; and it seems quite likely to the writer that the disease is lingering in our midst in this way.

"Tuberculosis in cattle is very prevalent not only here but in other States, and on account of its relation to the health of the people is the most important of all. This disease prevails much more extensively in Iowa than most of you or the people at large suppose. A forthcoming bulletin of the Iowa Experiment Station by the Veterinary Department will show that some herds have proven tuberculous to the extent of from 20 to 60 per cent. Others not suspected of being affected before the test have shown as high as 10 per cent. diseased. Many herds have, of course, proven free from disease. Of the total number tested by the State and Experiment Station about

15 per cent. have reacted to the tuberculin test. This is a large percentage, and would not be reached were all animals tested. The disease is not confined to any breed or class of cattle, but involves short-horns, polled Angus, Jerseys, scrubs, and others, as well as breeding herds, dairy cattle, private cows, and we may say animals kept under all conditions.

"Without doubt the disease is being quite extensively scattered over the country by breeders who have diseased herds, and this class of herds is quite numerous. Thus we see in this disease great danger to our cattle, and, as the disease may be communicated to man by the use of tuberculous meat or milk infected with the germs of the disease, great danger also to the public health. Here then is a great field for sanitary work, but, as our laws now are, the disease cannot be effectively dealt with. With proper laws much more would be possible.

"In relation to swine, the diseases of hog-cholera and swine-plague are important. You are well informed regarding the annual ravages of these affections. This loss could be and should be almost entirely prevented. With our present laws *this* is impossible. Strict quarantine against animals outside the State and of diseased herds within the State must be established. While these affections are not communicable to man, the economical feature is very important. While no figures are at hand showing the amount of yearly loss from these affections, it is apparent to all of you that it is sufficient to call for the application of the most thorough sanitary regulations.

"About the danger arising from the consumption of diseased animals and their products for food very much might be said, but we will only briefly discuss some of the more important points. Practically the people have no guarantee that they are using wholesome meats, milk, or butter. It is true we have some laws bearing on this point, but, while a penalty may attach to the selling of diseased meat, it is the duty of no one to learn whether diseased meats are sold or not, and if the butcher feels so disposed he can kill and sell the worst diseased animals to be found. When we go to the shop to purchase beef, we know not whether it came from a healthy carcass, a tuberculous animal, or one that was not in some other way rendered unfit for food. Pork, mutton, and veal also may or may not be wholesome. The only guarantee we have is the honesty of the dealer. The flesh if from an animal free from disease may, by being kept too long or in bad quarters, become unwholesome. As any animal may be killed and sold for food, so may the milk and butter from any animal be placed upon the market. As long as there is no inspection of our dairies and their products except the test for butter-fat in the milk, just so long may milk and butter infected with the germs of any disease be sold. It has been found that a number of diseases may be communicated in this way, for example, scarlet fever, diphtheria, typhoid fever, and tuberculosis. The last is by far the most important, since it has been proven that tuberculous cows often give infected milk. That milk from cows with non-tubercular udders may contain the tubercle bacilli has been shown by several investigators. Were tuberculosis a rare infection of milk cows, the danger from this source would not be great, but is by no means true of any part of the country. As before stated, the observations of veterinarians in this State have shown that many of our dairy herds are badly infected, as the following figures show. In one herd of dairy cows 30 out of 50 were diseased; in another 13 out of 30; this herd was supposed to be free from disease.

"It is not necessary in this report to enter into an argument to show that this disease *can be* communicated to man in the way previously indicated, nor enumerate other reasons why we should have thorough meat and milk inspection. That we have in this State no efficient inspection of dairy animals, their surroundings, etc., is well known to you all. In a paper read to this Association last year I endeavored to deal with the necessity for this inspection and discuss what it should consist of, and I will not further discuss the question here. At our last meeting the important subjects of government and city meat inspection were ably discussed in two papers, to which you are referred for further information.

"A few words regarding our part as sanitarians and we will end the report, which seems already long.

"In the sanitary work of the future the veterinarian must play a very important part. To a great extent he will be intrusted with the guarding of the public health. Inspectors of meat and of our dairies will be graduates of veterinary colleges. Not only will official veterinarians figure as sanitarians; the practitioner will give advice that will materially lessen the loss due to contagious and infectious diseases. Such advice will be asked for and paid for the same as other veterinary services. At the present time we can do much by assisting in the education of the public to a realization of the importance of sanitation as a safeguard to human life, and as a matter of economy. Hoping this subject of sanitation may be amply discussed by this body, this report is respectfully submitted."

On motion by Dr. Rich, with Dr. Stalker's second, the report was accepted. The discussions following were largely on the subject of tuberculin tests, and were indulged in by a goodly number of the members.

Through absence of the Chairman of the Committee on Collective Statistics no report was made.

Dr. Niles in reporting the conference of the committee appointed, with Dr. Hoskins, relative to publishing report and furnishing reprints to the members, said Dr. Hoskins would only print an abstract of the same. Moved by Dr. Gibson, duly seconded and carried, that the proceedings of the meeting be forwarded to Dr. H. and he should be privileged to publish such portion of it as he sees fit.

The change of Section 1 of Article 3 of our Constitution, as proposed at the annual meeting of 1894, to read: "Applicants for membership must be graduates of legally authorized veterinary colleges requiring a curriculum of three years, of not less than six months each; but this change shall not apply to matriculants prior to 1896." On motion of Dr. Whitbeck, seconded by Dr. Stalker, a vote was carried to adopt the change.

Drs. R. G. Rich and J. I. Gibson presented a petition asking for a change of Article 4 of By-Laws of I. S. V. M. A. refering to "dues," to read, "Each member on being admitted to membership shall pay the sum of two dollars, and shall annually thereafter pay one dollar in advance to the Association. Any member in arrears more than two years shall be suspended until said arrears are paid." Motion by Dr. Johnson, second by Dr. Gibson, voted and carried, that the Secretary have printed three hundred copies of the Constitution and By-Laws as now amended.

The election of officers for the ensuing year resulted in: Dr. L. U. Shipley, President, Sheldon, Ia.; Dr. R. G. Rich, First Vice-President, Fayette Ia.; Dr. T. A. Bown, Second Vice-President, Chariton, Ia.; Dr. J. E. Brown,

Secretary and Treasurer, Oskaloosa, Ia.; S. H. Kingery, Creston, Ia., J. I. Gibson, Denison, Ia., J. McBirney, Charles City, Ia., Board of Censors.

On motion of Dr. Niles, duly seconded, the meeting adjourned to meet in this city at the call of the President and Secretary.

JOHN E. BROWN,
Secretary.

